# Systemic Venous Inflow to the Liver Allograft to Overcome Diffuse Splanchnic Venous Thrombosis

**DOI:** 10.1155/2015/810851

**Published:** 2015-10-11

**Authors:** Cristian Lupascu, Tom Darius, Pierre Goffette, Jan Lerut

**Affiliations:** ^1^Department of Surgery, University of Medicine and Pharmacy “Gr.T.Popa”, University Hospital St. Spiridon, Iasi, Romania; ^2^Starzl Unit of Abdominal Transplantation, Cliniques Universitaires St. Luc, Université Catholique de Louvain (UCL), 1200 Brussels, Belgium; ^3^Department of Imaging, Unit of Interventional Radiology, Cliniques Universitaires St. Luc, Université Catholique de Louvain (UCL), 1200 Brussels, Belgium

## Abstract

Diffuse splanchnic venous thrombosis (DSVT), formerly defined as contraindication for liver transplantation (LT), is a serious challenge to the liver transplant surgeon. Portal vein arterialisation, cavoportal hemitransposition and renoportal anastomosis, and finally combined liver and small bowel transplantation are all possible alternatives to deal with this condition. Five patients with preoperatively confirmed extensive splanchnic venous thrombosis were transplanted using cavoportal hemitransposition (4x) and renoportal anastomosis (1x). Median follow-up was 58 months (range: 0,5 to 130 months). Two patients with previous radiation-induced peritoneal injury died, respectively, 18 days and 2 months after transplantation. The three other patients had excellent long-term survival, despite the fact that two of them needed a surgical reintervention for severe gastrointestinal bleeding. Extensive splanchnic venous thrombosis is no longer an absolute contraindication to liver transplantation. Although cavoportal hemitransposition and renoportal anastomosis undoubtedly are life-saving procedures allowing for ensuring adequate allograft portal flow, careful follow-up of these patients remains necessary as both methods are unable to completely eliminate the complications of (segmental) portal hypertension.

## 1. Introduction

Successful liver transplantation (LT) requires an adequate portal inflow; however, the reported incidence of PVT at moment of LT has been reported to vary between 2 and 35% [1–3]. In the eighties, even segmental portal vein thrombosis (PVT) was a contraindication to LT. The development of adapted surgical techniques, such as venous eversion thrombectomy, vascular interposition grafts, and use of portal vein (PV) collaterals, allowed us to perform successfully LT in such patients [1, 2, 4, 5]. Diffuse thrombosis of the venous splanchnic system (DSVT), however, still remained for a long time a technical contraindication to the transplant procedure leading finally to the introduction of combined liver-intestinal transplantation as the ultimate solution to overcome this difficult situation [6].

Starzl originally reported in 1973 about the use of cavoportal hemitransposition (CPHT) in the study of the pathophysiology of systemic venous flow on liver function and, later on, in the treatment of glycogen storage disease [7, 8]. His originally described technique was modified in 1998 by Tzakis et al. implying an end-to-end anastomosis between the native inferior vena cava (IVC) and the graft PV or a side-to-end anastomosis between both vessels followed by a calibration or clipping of the IVC lumen [9]. The renoportal anastomosis (RPA), first described by Sheil et al. in 1997 [10], was also modified by Azoulay and Kato using a venous interposition graft between RV and allograft PV [11, 12]. Although both procedures aim to procure a sufficient allograft PV inflow, they are unable to clear (completely) splanchnic portal hypertension [13]. Long-term follow-up of such procedures has been rarely reported in the literature explaining also why both procedures did not gain widespread use within the transplant community. Our study focuses on both CPHT and RPA as a means to secure the allograft portal inflow and the analysis of the technical feasibility as well as long-term consequences of these procedures.

## 2. Patients and Methods

Between 2000 and 2010, five liver recipients (1, 4%; *n* = 352) presented with diffuse splanchnic venous thrombosis at LT. Their demographics are summarized in Table 1. Indications for LT were Budd-Chiari syndrome (*n* = 1), HCV cirrhosis (*n* = 1), familial amyloidotic polyneuropathy (*n* = 1), and hepatic failure secondary to radiation-induced biliary cirrhosis after previous cholangiocarcinoma (CHCA) treated with left liver and pancreaticoduodenal resection (*n* = 2). The two latter patients also had HA thrombosis. One other patient had a splenectomy and splenorenal shunt 9 years before LT; this shunt was thrombosed at time of LT. All patients had advanced liver failure associated with symptomatic portal hypertension. Doppler ultrasound, coeliomesenteric angiography, and magnetic resonance or multislice computer tomography confirmed the extended splanchnic thrombosis.

Four patients underwent a CPHT with complete diversion of the IVC flow towards the allograft PV using a side-to-end anastomosis, directly (*n* = 2) or with the use of a venous iliac graft interposition (*n* = 2) from the same donor. In one patient, presenting a combined PV and HA thrombosis, the allograft arterialization was done using an interposition iliac arterial graft between iliac artery and allograft HA; venous inflow was restored using a venous iliac graft between the left renal vein and the graft PV. Postoperative management was done according to standard protocol. Immunosuppressive therapy was initiated with tacrolimus and methyl-prednisolone (4 days) and continued afterwards with tacrolimus monotherapy. None of the patients needed treatment for acute cellular rejection. Doppler ultrasound was performed daily in the intensive care unit and twice weekly during the first 3 weeks after transplantation. Post-LT anticoagulation was used in one patient only.

## 3. Surgical Technique

**Figure 1:**
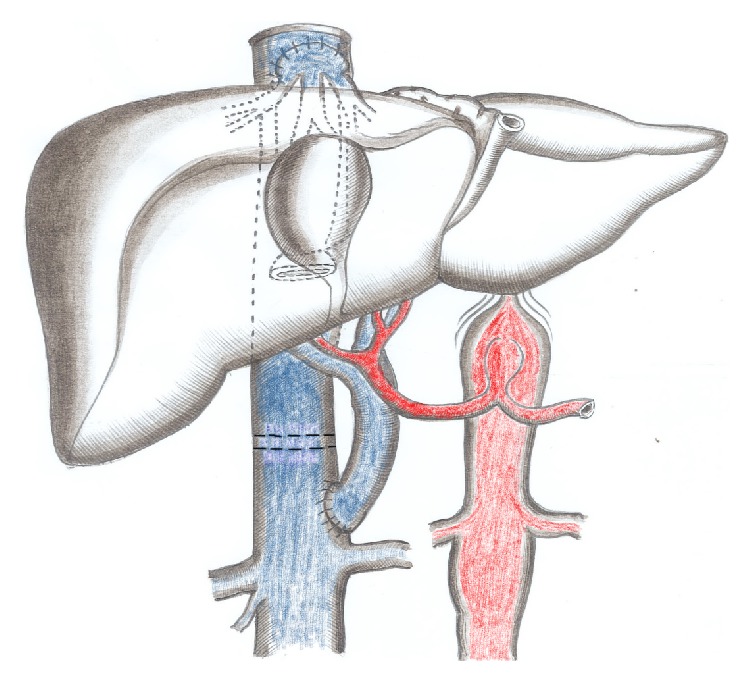
Side-to-end cavoportal hemitransposition with complete diversion of the IVC flow to the allograft and side-to-side cavocavostomy.

**Figure 2:**
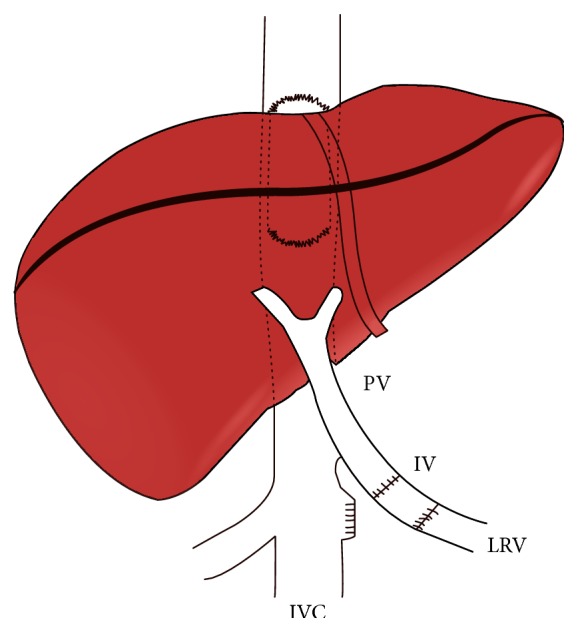
Side-to-side cavocavoplasty and end-to-end renoportal anastomosis with iliac vein graft interposition (portal vein; IV: iliac vein graft; LRV: left renal vein; IVC: inferior vena cava).

**Figure 3:**
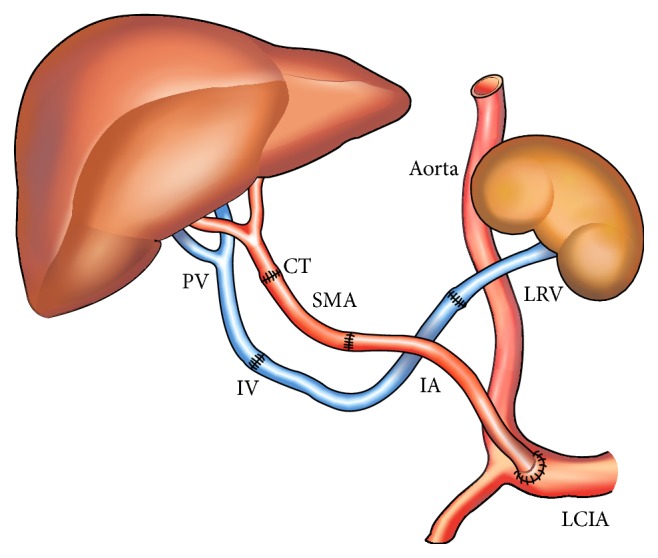
Complex reconstruction of portal and arterial flow to the allograft. Portal flow: end-to-end renoportal anastomosis with iliac vein graft interposition (PV: portal vein; IV: iliac vein graft; LRV: left renal vein); arterial flow: superior mesenteric artery (SMA) and iliac artery (IA) grafts (same donor) interposed between graft celiac trunk (CT) and left common iliac artery (LCIA).

IVC sparing hepatectomy was performed in all patients without the use of venovenous bypass. Four patients received a whole liver graft and one patient a reduced liver graft (segments I, IV–VIII). The graft was implanted using a large laterolateral cavocavostomy. Side-to-end CPHT between the infrahepatic IVC of the recipient and donor PV was performed four times. This anastomosis was done directly (*n* = 2) or using a venous iliac extension graft (*n* = 2). The infrahepatic caval flow was completely interrupted, after liver reperfusion, just above the level of the cavoportal anastomosis, using a double stapling device (Figure 1), and preventing thereby the diversion of blood flow from the liver allograft. One of the two patients with previous radiotherapy underwent an end-to-end anastomosis between left RV and allograft PV, using an iliac vein interposition graft (Figures 2 and 3) as the presence of hemorrhagic, thickened peritoneum around the IVC due to the fact that prior radiation induced peritoneal injury made the realization of the CPHT impossible; the problem was “solved” using an interposition venous graft between recipient left RV and allograft PV.

In three patients (pat. numbers 1, 2, and 4) the arterialization of the allograft was done anastomosing donor and recipient HAs. Both cases, with previous radiotherapy for CHCA, presented with a hepatic artery and celiac trunk thrombosis. The arterial flow to the graft was provided by an end-to-end anastomosis between the celiac trunk of the graft and the recipients' right common iliac artery using an iliac artery graft interposition (pat. 3) and an interposition conduit made of the superior mesenteric and iliac arteries between the donor celiac trunk and the recipients' left common iliac artery (pat. 5) (Figure 3). All complex portal and arterial reconstructions were placed in retrogastric and prepancreatic position.

## 4. Results

Graft portal reperfusion using systemic venous inflow could be restored successfully in all five cases. Median surgical time was 840 minutes (range 620−1260), and median cold ischemia time was 1080 minutes (range, 455–1195); median warm ischemia time was 45 minutes (range 23–77). The median autotransfusion and colloid administration were, respectively, 3028 mL (range 864–3500) and 3500 mL (range 2800–11600) (Table 2). Initial graft function was adequate in three patients. Primary nonfunction, observed in the patient with RPA (pat. nr. 5), needed re-LT after 5 days, using a reduced liver graft (segments I–IV) from a deceased donor. Initial poor function of the graft was observed in one patient with previous irradiation (pat. number 3). Both patients required surgical revision day 1 after LT because of intraperitoneal bleeding (Table 2).

Recurrent PVT was diagnosed in patient 5 two days after LT; he also needed early revision for early HA thrombosis and hepatic failure occurred (Table 3). Intraoperative liver biopsy revealed hepatic necrosis. Early IVC infrarenal thrombosis was diagnosed in the other patient with prior irradiation (pat. 3).

After LT, all patients developed transient ascites and varying degrees of transient renal dysfunction; two needed renal support therapy. One patient developed spontaneous bacterial peritonitis after LT (pat. 3). Transient cholangitis was observed in patient 4.

Two patients experienced severe gastrointestinal bleeding, respectively, 21 and 24 months after LT. One of them had an almost lethal bleeding due to an eroded varix at the hepaticojejunostomy. In this patient normal portal flow was restored after interventional radiologic stenting of an anastomotic stenosis of the cavoportal anastomosis. One patient needed urgent surgical hemostasis, splenectomy, and gastric devascularisation because of an upper GI bleeding. Interestingly the patient who underwent pretransplant splenectomy and splenorenal shunt did not develop any problem after LT.

After a median follow-up was 58 months (range, 3 weeks–130 months), three patients are well and alive. The two early deaths concerned both CHCA patients 3 and 5 which presented with secondary biliary cirrhosis following liver and pancreas resection and local irradiation. Patient 3 died two months after LT due to hepatorenal dysfunction and fungal sepsis. Patient 5 (who was retransplanted) died 18 days after the first LT due to MOF and combined HA and PV thrombosis. The three surviving patients are in a very good condition 59, 119, and 130 months after LT. Portal inflow was normal on ultrasound, MRI, or CT angiograms. They also had normal liver and gastrointestinal functions.

## 5. Discussion

Portal vein thrombosis, present in 2 to 35% of liver recipients, is an unfavorable condition to perform LT; however, nowadays most patients with PVT are amenable to conventional LT [1, 2]. Pathophysiological factors associated with PVT include male gender, previous treatment of portal hypertension (sclerotherapy, TIPSS, shunt surgery, splenectomy, and gastric devascularisation), Child-Turcotte-Pugh C status, and type of cirrhosis [2]. In such cases liver revascularization is almost always possible using eversion venous thrombectomy, interposition of venous iliac graft between donor portal and recipient superior mesenteric veins, or anastomosis of PV to a large venous collateral such as the coronary vein [1–4]. Patients with diffuse splanchnic venous thrombosis (DSVT) represent in contrast a great challenge for the transplant surgeon as none of the abovementioned techniques is able to solve the problem. Different solutions have been proposed in this transplant setting. Arterialization of the PV using, for example, an interposition graft between recipient aorta and graft PV is limited in its application as the proper calibration of the arterialization and the arterial hepatic hyperperfusion syndrome usually seriously compromise the outcome [14–17]. The combined liver-intestinal transplantation has been proposed as another treatment modality. The advantage of this type of transplantation, namely, to eliminate the splanchnic congestion in counteracted by its high morbidity and still poor long-term outcome (3-years patient survival of only around 50% at experienced centers) [6]. The major shortcomings of both abovementioned procedures led to the more frequent application of the less invasive CPHT procedure. Some 80 cases have been reported in the literature [18–21]. CPHT implies a side-to-end anastomosis, using either a direct approach or an interposition graft, between the recipient IVC and the donor PV. Azoulay et al. proposed a controlled clipping of the IVC in order to preserve caval as well as hepatopetal flows [11]. We further refined the technique in order to avoid well-known thrombotic venous complications. This technique combines IVC sparing hepatectomy and large cavocaval and cavoportal anastomoses flush to both sides of the IVC interrupting stapling line. By doing so excellent caval and portal flow can be obtained at both sides of the interrupted IVC, a condition that allows for avoiding the formation of (potentially lethal) clots and thrombosis in an otherwise excluded part of the interrupted IVC [8] (Figure 1). When LT-CPHT procedure is impossible, RPA represents an alternative technique to ensure systemic venous allograft inflow. This technique has the advantage that it does not interrupt IVC flow nor disconnects existing portosystemic venous communications and that it allows for retaining the spleen, an important feature in immunosuppressed patients (Figure 2). Azoulay and Kato advocated that the RPA should be the strategy of choice in liver recipients having preexistent spontaneous or surgically constructed splenorenal shunts. RPA has the advantage that left RV and PV are well matched and coaxial venous structures; moreover, the physiological retrohepatic IVC flow, which is devoided only from the left renal venous inflow, remains preserved [11, 12].

The fact that many patients are surviving after a LT-CPHT procedure with an excellent graft function (as showed also in three of our patients) indicates that the splanchnic hepatotropic factors reach, in the absence of direct perfusion of the liver by splanchnic derived blood, the liver allograft through extra-anatomical portocaval shunts which finally find their “end destination” trough the CPHT [13, 18, 19]. The main drawback of CPHT (and of RPA) lies in its' inability to adequately decompress the splanchnic venous system [13, 18]. Indeed the LT-CPHT procedure transforms the condition of DSVT and end-stage liver disease into the condition of DSVT with healthy liver only. Although the (scarce) clinical experience with this procedure allowed us to observe that signs of portal hypertension such as variceal bleeding and ascites often gradually lower or even disappear over a period of months after LT, many patients needed reinterventions for (severe) gastrointestinal bleeding (as showed in two of our patients). The long-term effects of deriving systemic venous flow towards the liver allograft are currently unknown, so life-long follow-up of these (rare) patients is necessary in order to draw definitive conclusions regarding the final impact of CPHT and RPA in liver recipients presenting with DSVT and also to timely take even prophylactic, medical, endoscopic, and surgical therapeutic decisions when (bleeding) complications occur.

Our analysis also showed the limits of the LT-CPHT procedure. Clinical studies have indicated that patients who have previously undergone radiation therapy for malignancies are at increased risk for developing vascular diseases and that their risk is further amplified in the presence of traditional cardiovascular risk factors [22, 23]. LT recently emerged as an effective treatment for patients with localized lymph node negative, irresectable hilar CHCA after neoadjuvant chemoradiotherapy and after combined radiotherapy and en bloc hepatectomy-duodenopancreatectomy for early stage hilar CHCA [24, 25]. Both patients in our series who underwent the latter procedure died after LT-CPHT and RPA procedures. They not only presented with DSVT but also with HA thrombosis and pronounced radiation-induced peritoneal injury making the hepatic and abdominal dissection very difficult and even hazardous. The impossibility to use the native IVC for CPHT in one patient obliged us to perform a RPA to ensure portal allograft inflow. Both patients also needed complex arterial reconstructions and early surgical revision because of recurrent bleeding and vascular thrombosis. The pre-LT radiation induced peritoneal injury represented also an additional risk factor for primary nonfunction or initial poor function of the graft due to the prolonged ischemia times resulting from the encountered intraoperative technical difficulties. Such major technical difficulties have indeed been encountered in the CHCA liver transplant series from the Mayo clinic [24, 25].

In conclusion, diffuse splanchnic venous thrombosis is no longer an absolute contraindication for LT. CPHT and RPA are salvage procedures which may allow favorable outcome in these patients; they represent a very valuable alternative to the more aggressive combined liver-intestinal transplantation. Radiation induced peritoneal injury represents an additional risk factor in LT alongside an extensive splanchnic venous thrombosis.

As CPHT and RPA are unable to decompress adequately the splanchnic venous system, these patients should have a tight life-long follow-up looking especially at the risk to develop severe gastrointestinal bleedings.

## Figures and Tables

**Table 1:** Recipient pretransplant data.

	Patient 1	Patient 2	Patient 3	Patient 4	Patient 5	Median
Age (yrs)	56	42	43	36	69	43

Gender	Female	Male	Male	Female	Male	

Indication for LT	Budd-Chiari syndrome	HCV cirrhosis	Hepatic failure due to radiation-induced biliary cirrhosis	FAP	Hepatic failure due to radiation-induced biliary cirrhosis	

Blood type	O	A	O	A	O	

CTP	A	C	A	A	B	

UNOS sc	3	2	3	3	3	3

MELD sc	22	18	11	9	15	15

Thrombosis risk factor	—	—	Neoplasia + radiotherapy	—	Neoplasia + radiotherapy	

GI bleed	—	Varices	—	—	Varices	

Pre-LT surgery	—	Splenectomy and splenorenal shunt	Le hepatectomy with HA and PV reconstruction and PDR for CHCA	—	Le hepatectomy and PDR for CHCA gastrostomy	

**Table 2:** Transplant procedure data.

	Patient 1	Patient 2	Patient 3	Patient 4	Patient 5	Median
VVB	No	No	No	No	No	
Implantation of IVC	Side-to-side	Side-to-side	Side-to-side	Side-to-side	Side-to-side	
Type of liver graft	Whole	Whole	whole	whole	Reduced (S5-8)	
Type venous graft	Iliac vein	Iliac vein	—	—	Iliac vein	
Type arterial graft	—	—	Iliac artery	—	Iliac + SM artery	
CPHT type/RPA	Side-to-end	Side-to-end	Side-to-end	Side-to-end	RPA end-to-end	
Biliary anastomosis	Roux-Y HJ	Roux-Y HJ	Roux-Y HJ	Roux-Y HJ	Ext biliary drainage Day 1 HJ	
Blood transfusion mL	0	1000	83000	0	2510	0
Autotransfusion	864	3028	3481	900	3500	3028
Platelets unit	0	9	11	0	0	0
Colloids	360	5200	11600	2800	3500	3500
Operative time (min)	620	840	1260	634	1080	840
CIT (min)	675	1080	1195	455	1080	1080
WIT	40	45	23	50	77	45
Anhepathy (min)	385	115	Data NA	98	Data NA	—

(CPHT: cavoportal hemitransposition, CIT: cold ischemia time, RPA: renoportal anastomosis, SM: superior mesenteric, and WIT: warm ischemia time).

**Table 3:** Outcome after LT for diffuse splanchnic venous thrombosis.

	Pat 1	Pat 2	Patient 3	Pat 4	Pat 5
IS	TAC	TAC	TAC	TAC	TAC

Re-LT	No	No	No	No	Yes (PNF)

Post-LT surgery	Splenectomy, gastric devascularization Redo Roux-YHJ	—	d1:bleed d32 evisceration d43 parietal prosthesis	splenectomy, and gastric devascularization and Roux-Y HJ	d1:bleed Roux-YHJ parietal prosthesis d11:HA thrombectomy

Indication reoperation	GI bleed from varix at HJ	—	bleed parietal infect	GI bleed from varices	bleed allograft failure

Acute rejection	No	No	No	No	No

Infection	No	No	Parietal and ascites CMV fungal sepsis	Cholangitis	No

IPF or PNF	No	No	IPF	No	PNF

IVC thrombosis	No	No	Yes	No	No

Cause of death	—	—	Septic shock MOF	—	PVT; HAT; MOF

Status (mo)	Alive (130)	Alive (109)	Death (2)	Alive (58)	Death (0,5)

(GI: gastrointestinal, HA: hepatic artery, HAT: hepatic artery thrombosis, HJ: hepaticojejunostomy, IPF: initial poor function, IS: immunosuppression, PNF: primary nonfunction, PVT: portal vein thrombosis, and TAC: tacrolimus).

## Abbreviations Used in the Text

CHCA: Cholangiocarcinoma
CPHT: Cavoportal hemitransposition
CT: Celiac trunk
DSVT: Diffuse splanchnic venous thrombosis
HA: Hepatic artery
Hat: Hepatic artery thrombosis
GI: Gastrointestinal
IA: Iliac artery
IPF: Initial poor function
IV: Iliac vein
IVC: Inferior vena cava
LCIA: Left common iliac artery
LT: Liver transplantation
LRV: Left renal vein
PDR: Pancreatoduodenectomy resection
PNF: Primary nonfunction
PV: Portal vein
PVT: Portal vein thrombosis
RPA: Renoportal anastomosis
SMA: Superior mesenteric artery.
